# Truffle Microbiome Is Driven by Fruit Body Compartmentalization Rather than Soils Conditioned by Different Host Trees

**DOI:** 10.1128/mSphere.00039-21

**Published:** 2021-08-11

**Authors:** Dong Liu, Jesus Pérez-Moreno, Xinhua He, Roberto Garibay-Orijel, Fuqiang Yu

**Affiliations:** a The Germplasm Bank of Wild Species, Yunnan Key Laboratory for Fungal Diversity and Green Development, Chinese Academy of Sciences, Kunming, Yunnan, China; b Colegio Postgraduados, Campus Montecillo, Edafología, Texcoco, México; c Department of Land, Air, and Water Resources, University of California—Davis, Davis, California, USA; d Instituto de Biología, Universidad Nacional Autónoma de México, Ciudad de México, México; University of Wisconsin—Madison

**Keywords:** truffles, community structure, microbial ecology, microbial networks, microbiome drivers

## Abstract

Truffles are among the most expensive edible mushrooms; their value is worth billions of U.S. dollars annually in international markets. They establish ectomycorrhizal symbiotic relationships with diverse host tree roots and produce hypogeous ascomata. Their whole life cycle is closely related to their associated microbiome. However, whether truffle-associated compartments or host tree rhizospheres are the vital driver for truffle ascomata microbiome is unclear. To identify and compare fungal and bacterial communities in four truffle-associated compartments (*Tuber indicum*: bulk soil, adhering soil to peridium, peridium, and gleba) from three host trees, we sequenced their ITS (fungal) and 16S (bacterial) ribosomal DNA using the Illumina MiSeq high-throughput platform. We further applied the amplicon data to analyze the core microbiome and microbial ecological networks. *Tuber indicum* microbiome composition was strongly driven by its associated compartments rather than by their symbiotic host trees. Truffle microbiome was bacteria dominated, and its bacterial community formed a substantially more complex interacting network compared to that of the fungal community. The core fungal community changed from Basidiomycota dominated (bulk soil) to Rozellomycota dominated (interphase soil); the core bacterial community shifted from *Bacteroidetes* to *Proteobacteria* dominance from truffle peridium to gleba tissue. Especially, at the truffle and soil interphase, the niche-based selection of truffle microbiome was verified by (i) a clear exclusion of four bacterial phyla (*Rokubacteria*, *Nitrospirae*, *Chloroflexi*, and *Planctomycetes*) in gleba; (ii) a significant decrease in alpha-diversity (as revealed by Chao 1, Shannon, and Simpson indices); and (iii) the complexity of the network substantially decreased from bulk soil to soil-truffle interphase and further to the peridium and gleba. The network analysis of microbiome showed that the microbial positive interactions were higher in truffle tissues than in both bulk soil and peridium-adhering soil and that *Cupriavidus*, *Bradyrhizobium*, *Aminobacter*, and *Mesorhizobium* spp. were the keystone network hubs in the truffle gleba. This study provides insights into the factors that drive the truffle microbiome dynamics and the recruitment and function of the microbiome components.

**IMPORTANCE** Currently, the factors that drive the microbiome associated with truffles, the most highly prized fungi in the world, are largely unknown. We demonstrate for the first time here that truffle microbiome composition is strongly driven by associated compartments rather than by symbiotic host trees. The truffle microbiome was bacteria dominated, and its bacterial community formed a substantially more complex (with the higher numbers of nodes, links, and modules) interacting network compared to that of the fungal community. Network analysis showed a higher number of positive microbial interactions with each other in truffle tissues than in both bulk soil and peridium-adhering soil. For the first time, the fungal community structure associated with truffles using high-throughput sequencing, microbial networks, and keystone species analyses is presented. This study provides novel insights into the factors that drive the truffle microbiome dynamics and the recruitment and function of the microbiome components, showing that they are more complex than previously thought.

## INTRODUCTION

As the most famous ectomycorrhizal fungi, truffles establish symbiotic relationships with a wide variety of host trees, such as *Pinus* and *Quercus* (1), playing vital roles in maintaining healthy and sustainable forest ecosystems through their interactions with soil microbiome. Studies have explored the microbiome composition of some truffles and the physiology of associated trees by studying a single host species. For example, *Tuber indicum* can shape microbiome in ectomycorhizophere soil in a *Pinus armandii* forest, while the influence the host physiology in a *Quercus acutissima* stand (2) and metabolic profiles in a *Q. aliena* stand (3). The long-term stability of microbiomes is crucial since the persistent occurrence of beneficial microbes and their associated functions ensure host health and fitness. In general, it is known that ectomycorrhizal community compositions are primarily structured by host trees, regardless of the soil environments. In addition, there is a preference of ectomycorrhizal fungi for particular host tree species (4). Due to the fact that plant root-associated microorganisms feed primarily on plant rhizodeposits, differential C allocation, released root exudates, and consequently nutrient availability by host trees are crucial environmental factors that may drive microbial communities (5). Root exudates e.g., fatty acids, sterols, and sterol esters, are of paramount importance in the selection of ectomycorrhizal symbionts by differentially promoting the mycelial growth of some fungi (6). However, if the soils conditioned by different host trees drive the microbiome associated with truffles has not accurately been evaluated.

On the other hand, a differentiation of the ascomata-associated microbiome might be developed between peridium and gleba due to the process of ascomata development. Colonization of the gleba tissue by soil microbiome may occur in advance to the differentiation of the truffle peridium when the primordium (a yellowish mycelial pellet) is entirely in contact with the soil (7). Then, certain microbes are gradually trapped in the ascocarpic tissue during the development of primordium and thus protected from soil exchange by the outer part of peridium. During this process, a part of the microbiome can be “sieved” and excluded from colonization, as demonstrated by a significant decrease in bacterial diversity in the ascomata compared to the bulk soil (8). Indeed, the selection of certain microbes is strongly related to their capacities to use particular carbon compounds occurring in mycosphere exudates (8). Several studies have shown that truffle ascomata are densely colonized by bacteria (9–11) and explored their roles in ascomata maturation (7), aroma formation (12, 13), and potential nitrogen fixation (7, 14). However, the coexisting fungi have received little attention, and whether the ascomata fungal communities are separated from those in the bulk soil is currently unknown. In order to comprehensively understand the truffle-associated niche and the selection of a specific microbiome, the interphase between truffle and soil should not be ignored. During ascomata development, the peridium is formed around isolating it from soil (7). Peridium-adhering soil is a microhabitat hot spot for microbiome activity in terms of exchanging nutrients and signals (15–17). Thus, identifying soil-truffle interphase microbiome could deepen our understanding of truffle ecology.

In a truffle-associated microhabitat, microbiomes are dynamic and highly diverse, making them challenging to understand (15, 17, 18). The identification of core microbial taxa (which drive community composition and function irrespective of their abundance) in soil, truffles, and/or their interphase could provide insights into microbiome interactive roles, microbial consortia, and resistance to external environmental disturbances (19, 20). Molecular ecological network analysis (MENA) has been widely used as a powerful way to reveal the complicated community assembly and microbial taxa under a diverse range of soil and plant microbiome interactions (21–23). As positive cooperation or negative competition, such interactions can be described by network models (23–25), in which individual nodes within a network represent microbial taxa (OTUs) and the edge bridging two nodes refers to their interrelationships (positive or negative) (23–25). MENA is also applied to identify keystone taxa that are important for maintaining community structure and function (26–28). In addition, spatial heterogeneity can drive the distribution of keystone taxa in any environment. For example, different keystone taxa may function individually, while multiple taxa with similar functioning (e.g., nitrogen fixation) may form a keystone guild and alter the structure and dynamics of their ecosystems (28). In the soil microbiome, bacterial and fungal keystone taxa have been computationally inferred/identified using network scores (28–30). For a range of taxa, it has been shown that keystone taxa identified using statistical tools indeed have an impact on the composition and performance of the microbiome (21, 28, 30, 31). Microbial keystone taxa have been widely identified in agroecosystems (32–34), but not in forest ecosystems, especially in those having ectomycorrhizal woody trees, despite their great ecological and functional importance. A compartmentalization of biogeochemical cycles has been found in truffle ascocarp peridium and gleba (7, 35). As a result, knowledge of how keystone taxa could respond to ectomycorrhizal fungi or their fungal ascomata compartments could increase our understanding of their ecological value.

The central idea of the present study was to understand the effects of soils conditioned by different host trees and truffle-associated compartments (bulk soil, soil adhered to truffle peridium, peridium to gleba) on the variation in truffle microbiome by including its interphase that links the outer and inner parts of the truffle. Specific objectives were to explore (i) the dominance, composition, and keystone species of bacteria and fungi in truffle tissues and its inhabiting soils; (ii) microbial alpha taxonomic and their phylogenetic diversity; and (iii) the relative importance of host tree and truffle-associated compartments, using bacterial and fungal network analyses. In doing so, we used 16S and ITS rRNA gene sequencing to explore the taxonomic and/or phylogenetic diversity of microbial communities. We employed a random “matrix” theory analysis (based on amplicon data) to investigate the core microbiome and microbial ecological networks. In initial truffle ectomycorrhizal developing stage, the number of bacterial species were more than 5 times higher than that of fungal species, in both ectomycorrhizospheric soil and ectomycorrhiza (1). Based on a recent study by Liu et al. (36) showing that the bacterial OTUs in mushroom and truffle compartments are higher than the fungal OTUs, we hypothesized that the OTUs in the four truffle-associated compartments would be substantially higher in bacteria than in fungi (H1). Because of the existence of interphases (peridium and its adhering soil) between soil and truffle, there would be a clear selection of microbiome by soil and peridium leading to a significant decrease in microbial diversity, evenness, and richness (H2). Finally, under such selecting influence, the truffle microbiome composition would tend to be compartment driven (H3). Moreover, considering microbiome diversity and composition changes, we finally hypothesized that the OTU numbers and their associations would be more complex in a bacterial network than in a fungal network and that such network complexity would gradually decrease from the bulk soil, to the soil-truffle interphase, and finally to the gleba tissue (H4).

## RESULTS

### Differences in microbial richness and diversity indices.

**FIG 1:**
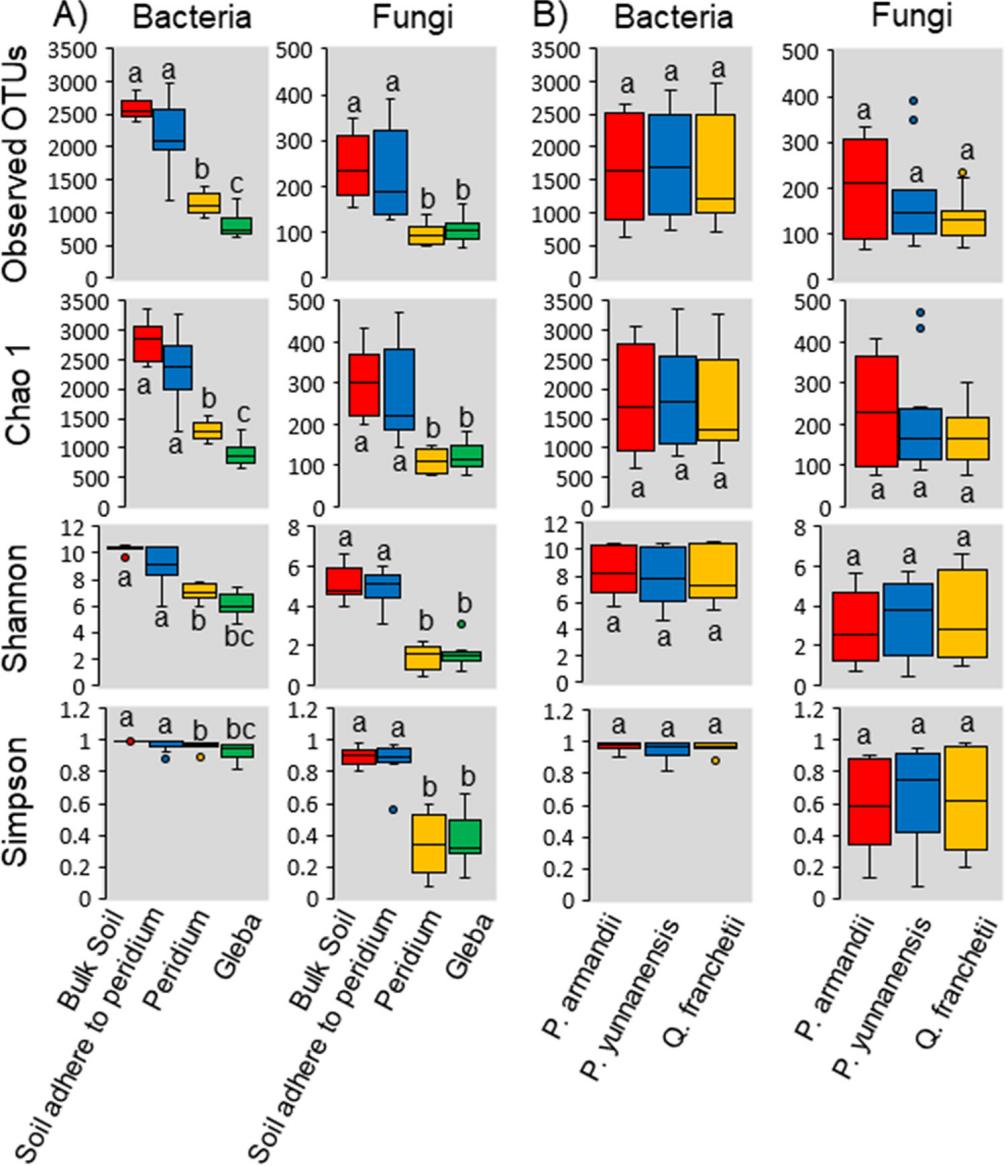
Observed OTUs and bacterial and fungal diversity indices of *Tuber indicum* ascomata and adhering soil. (A) Effect on soil and ascomata compartments; (B) effect on host trees. Alpha-diversity indices based on microbial richness (Chao 1 index), diversity (Shannon index), and evenness (Simpson index). For individual index boxes, significant differences for compartments or host trees and *post hoc* grouping are indicated by lowercase letters (Tukey HSD, *P < *0.05).

The numbers of total reads pre- and postfiltering were 1,611,239 and 1,465,346 for the V4 region of the 16S rRNA gene and 1,411,568 and 1,334,610 for the fungal ITS 1 region, respectively. The numbers of bacterial 16S rRNA sequences were higher in truffle tissue (average ± the standard deviation; peridium, 43,472 ± 19,930; gleba, 41,277 ± 12,129) than in soils (bulk soil, 40,463 ± 6,220; soils adhered to the peridium, 37,605 ± 6,771). In contrast, the numbers of fungal ITS sequences were higher in soils (bulk soil, 35,965 ± 3,103; soils adhered to the peridium, 36,172 ± 3,614) than in truffle tissues (peridium, 37,309 ± 6,650; gleba, 38,845 ± 4,699) (Fig. 1A). Microbial alpha-diversity indices (Chao 1, Shannon, and Simpson) of *Tuber indicum* ascomata growing in soils conditioned by three host trees did not differ (*P > *0.05; Fig. 1B). In contrast, when comparing the four ascomata compartments, we found that the three indices were all significantly higher in both bulk soil and adhering soil to peridium than in the truffle’s peridium and gleba (*P < *0.05; Fig. 1A). The Chao 1, Shannon, and Simpson indices in all the four different compartments (bulk soil, adhering soil to peridium, peridium, and gleba) of *Tuber indicum* were significantly higher for bacteria than for fungi.

### Variations in bacterial community structures.

In order to compare the beta-diversity of bacterial communities, nonmetric multidimensional analyses (NMDS) were performed. These analyses distinguished four truffle-associated compartments (analysis of similarities [ANOSIM], *R *=* *0.90, *P = *0.001), whereas the influence of host tree on bacterial community was not significant (ANOSIM, *R *=* *0.18, *P = *0.15). The difference in bacterial community structure, as shown by diverging of the shape, enlarged from bulk soil, soil adhered to peridium, and then peridium to gleba (see Fig. S1 in the supplemental material). Weighted UniFrac measurements in all truffle-associated compartments were significantly different for bacterial communities (Table 1), indicating that the phylogenetic structure of the bacteria was also related to compartmentalization. In contrast, the soils conditioned by different host trees did not influence their microbial community structure in truffle-associated compartments (Table 1).

**TABLE 1:** Bacterial community phylogenetic composition UniFrac comparison between truffle compartments and host tree soil^*a*^

Group 1	Group 2	Bacteria	
		F statistic	*P*
All within group	Between group	(0.2929)	0.7697
Between group	Pa vs Py	0.6035	0.5464
	Pa vs Qf	(0.8669)	0.3863
	Py vs Qf	0.2534	0.8001
			
All within group	Between group	(13.8434)	0.0000
Between group	P vs SP	1.9867	0.0474
	P vs BS	(5.6141)	0.0000
	P vs G	7.7697	0.0000
	SP vs BS	6.2622	0.0000
	SP vs G	(2.0313)	0.0427
	BS vs G	(8.5964)	0.0000

a Alternative hypothesis: group 1 mean = group 2 mean. Significance tests were performed using the F statistic. In group 2, abbreviations are as follows: Pa, *Pinus armandii*; Py, *Pinus yunnanensis*; and Qf, *Quercus franchetii* (for soils conditioned by host trees) and BS, bulk soil; SP, soil adhered to the truffle peridium; P, peridium; and G, gleba (for truffle compartments). Values in parentheses indicate negative values.

10.1128/mSphere.00039-21.1 FIG S1 Bacterial community compositions as indicated by unweighted NMDS plots of pairwise UniFrac community distance across the *Tuber indicum* recorded in four different ascocarp compartments and three different host trees. Each abbreviation is composed of “compartment/soil conditioned by host tree.” For truffle compartments, BS = bulk soil; SP = soil adhered to the truffle peridium; *P* = peridium; and G = gleba. For soil conditioned by host trees, Pa = *Pinus armandii*; Py = *Pinus yunnanensis*; and Qf = *Quercus franchetii*. Download FIG S1, DOC file, 0.2 MB. Copyright © 2021 Liu et al. 2021 Liu et al. https://creativecommons.org/licenses/by/4.0/ This content is distributed under the terms of the Creative Commons Attribution 4.0 International license.

### Common OTUs and distribution features of microbial community.

We built the bacterial phylogeny of common OTUs over the four truffle-associated compartments to assess their distribution and phylogenetic diversity. The number of common OTUs present in all compartments was 995 for bacteria and 157 for fungi (see Table S1 [files 1 and 2] and Fig. S2). For bacteria, all compartments were dominated by *Proteobacteria* (59.5% of all OTUs) and *Actinobacteria* (11.0%), followed by *Bacteroidetes* (7.2%), *Acidobacteria* (7.0%), *Firmicutes* (4.3%), *Chloroflexi* (3.3%), and *Planctomycetes* (2.0%) (see Table S1 [file 1] and Table S2). *Rokubacteria*, *Nitrospirae*, *Chloroflexi*, and *Planctomycetes* were only found in soils, but not in the peridium and gleba. In contrast, *Bacteroidetes* predominated in the peridium, while *Proteobacteria* was mainly in the gleba (see Fig. S3). *Sphingomonas*, *Afipia*, *Amycolatopsis*, Acinetobacter, *Bradyrhizobium*, *Cupriavidus*, and Pseudomonas preferred to colonize the gleba (>70% relative abundance). Fungi were dominated by Ascomycota (79.3%), followed by Basidiomycota (9.7%), Mucoromycota (3.7%), Mortierellomycota (1.1%), Chytridiomycota (0.6%), and Rozellomycota (0.4%). Fungal species belonging to Basidiomycota mostly occupied bulk soil, whereas taxa assigned to Rozellomycota preferred the soil adhering to peridium. In the peridium, six species (five from the phylum Ascomycota [*Cosmospora_gigas*, *Clonostachys_intermedia*, *Exophiala_cancerae*, *Phaecoacremonium_hungaricum*, *Penicillium_chermesinum*] and one from the phylum Basidiomycota [*Anthracoidea_aspera*]) had high relative abundances (average, 52%). Only three taxa—*Simplicillium_aogashimaense*, *unidentified_Rozellomycota*, and *Cutaneotrichosporon_cyanovorans* (∼65%)—showed a very high abundance in the gleba (see Table S1 [file 2] and Table S2).

10.1128/mSphere.00039-21.2 FIG S2 OTU numbers of bacteria (A) or fungi (B) shared or exclusive of four *Tuber indicum* compartments (BS = bulk soil; G = gleba; *P* = peridium; SP = soil adhered to peridium). The total number of shared OTUs bacteria and fungi is shown in the center. The OTUs exclusively present in each compartment are shown in different colors as follows: dark pink = peridium, blue = bulk soil, pink = gleba, and green = soil adhere to peridium, and all the possible intersections. Download FIG S2, DOC file, 0.4 MB. Copyright © 2021 Liu et al. 2021 Liu et al. https://creativecommons.org/licenses/by/4.0/ This content is distributed under the terms of the Creative Commons Attribution 4.0 International license.

10.1128/mSphere.00039-21.3 FIG S3 Phylogenetic tree of OTUs common to the four truffle-associated compartments based on analysis of 16S rRNA genes. Colors of stripes indicate different major phyla. The pie charts represent the relative abundance of each OTU, and the pie slice colors the distribution across compartments (bulk soil in red, soil adhered to the truffle peridium in green, peridium in blue, and gleba in blue). Download FIG S3, DOC file, 0.5 MB. Copyright © 2021 Liu et al. 2021 Liu et al. https://creativecommons.org/licenses/by/4.0/ This content is distributed under the terms of the Creative Commons Attribution 4.0 International license.

10.1128/mSphere.00039-21.4 TABLE S1 Supplementary OTU data sets and representative sequences. (A) Bacterial OTUs; (B) fungal OTUs; (C) sequence number; (D) ANOSIM test. Download Table S1, XLS file, 3.1 MB. Copyright © 2021 Liu et al. 2021 Liu et al. https://creativecommons.org/licenses/by/4.0/ This content is distributed under the terms of the Creative Commons Attribution 4.0 International license.

10.1128/mSphere.00039-21.5 TABLE S2 Relative abundances of the most abundant bacteria and fungi in *Tuber indicum* compartments. Download Table S2, DOC file, 0.1 MB. Copyright © 2021 Liu et al. 2021 Liu et al. https://creativecommons.org/licenses/by/4.0/ This content is distributed under the terms of the Creative Commons Attribution 4.0 International license.

### Distinct microbial networks and keystone taxa.

**FIG 2:**
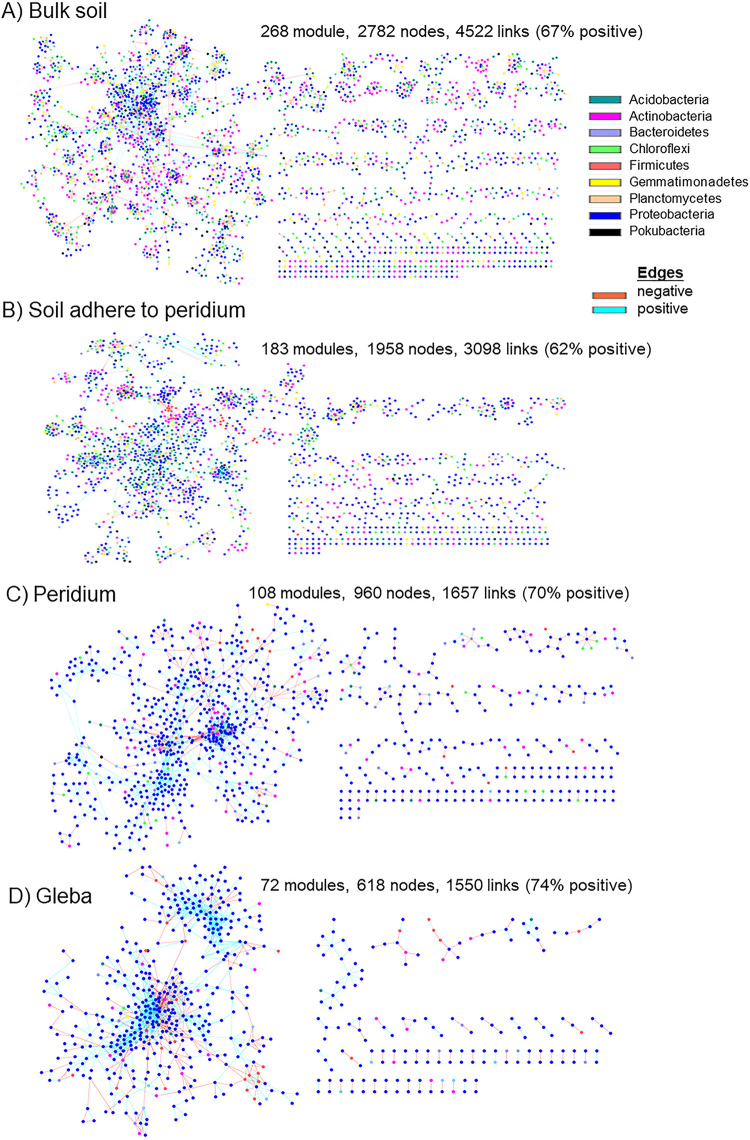
Network interactions of bacteria in truffle-associated compartments. Points represent OTUs, and the point colors indicate different major phyla. Solid lines represent relationships among nodes. A module is a cluster of highly interconnected nodes. Interpretation of the abbreviations is presented in Table 1.

**FIG 3:**
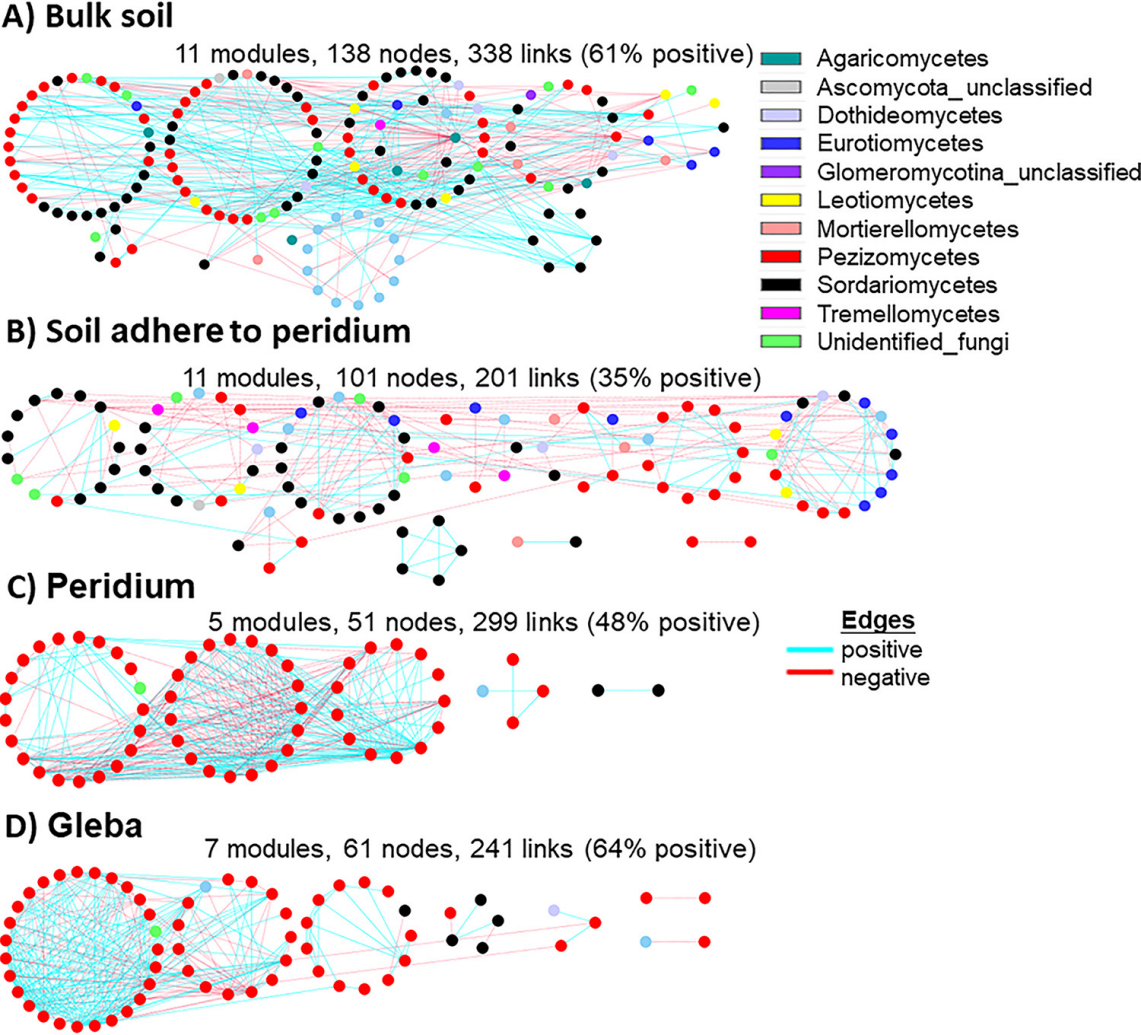
Network interactions of fungi in truffle-associated compartments. Nodes represent OTUs, and node colors indicate different major classes. Solid lines represent relationships among nodes. A module is a cluster of highly interconnected nodes. Interpretation of the abbreviations is presented in Table 1.

Eight ecological networks were constructed using the bacterial and fungal OTUs from the four truffle-associated compartments (Fig. 2 and 3). The networks showed positive or negative interactions among taxa, and the nodes were distinguished by different colors at the phylum level. The similarity thresholds of the networks ranged from 0.83 to 0.94 (Table 2), which were higher than those of most reported networks by this method (37, 38). The indices, such as average connectivity, average path length, average clustering coefficient and modularity, were all higher in the ecological network than in the random network (Table 2), indicating that the constructed networks from this study can be used for subsequent studies on the potential interactions among the bacterial and fungal communities based on their network characteristics such as modular and scale free (24, 39).

**TABLE 2:** Topological parameters of empirical molecular ecological networks (MENs) of microbial communities in truffle compartments and their associated random MENs^*a*^

Compartment	Empirical network (avg)							Random network (avg ± SD)		
	St	Node	Link	Modularity	GD	CC	K	GD	CC	Modularity
Bacteria										
BS	0.94	2,782	4,522	0.932	11.6	0.156	3.1	5.4 ± 0.02	0.003 ± 0.001	0.613 ± 0.003
SP	0.94	1,958	3,098	**0.938**	11.4	0.148	3.2	5.3 ± 0.03	0.004 ± 0.001	0.625 ± 0.003
P	0.91	960	1,657	0.821	7.80	0.126	3.5	4.6 ± 0.04	0.01 ± 0.01	0.57 ± 0.01
G	0.89	618	1,550	0.673	6.29	0.208	5.0	2.5 ± 0.06	0.25 ± 0.02	0.22 ± 0.01
										
Fungi										
BS	0.83	138	338	0.615	4.46	0.254	4.7	3.2 ± 0.05	0.07 ± 0.01	0.41 ± 0.01
SP	0.85	101	201	**0.639**	4.50	0.200	3.7	3.5 ± 0.08	0.04 ± 0.01	0.48 ± 0.01
P	0.89	51	299	0.176	2.60	0.366	10.9	2.1 ± 0.04	0.39 ± 0.02	0.16 ± 0.01
G	0.87	61	241	0.281	3.17	0.287	7.8	2.4 ± 0.05	0.25 ± 0.02	0.22 ± 0.01

a St, similarity threshold; network size, the number of nodes; K, connectivity among nodes; GD, geodesic distance between nodes; CC, clustering coefficient of nodes; BS, bulk soil; SP, soil adhered to the truffle peridium; P, peridium; G, gleba. Boldfacing indicates the maximum values of modularity for the bacterial or fungal networks.

The bacterial network was more complex than that of fungi, containing ca. 10 to 15 times more nodes and links, a comparative higher modularity, and average geodesic distance between nodes but a comparative lower average clustering coefficient of nodes and average connectivity between nodes (Table 2). When considering the aggregate or loose structure of the network (as shown by the distance and connection between nodes), the average geodesic distance (GD) was lower, whereas the average clustering coefficient and connectivity were generally higher in truffle (P and G) compared to soil (BS and SP) networks (Table 2; *P < *0.05). A similar trend in all the parameters tested above in the empirical networks was also observed between the two soil compartments of bulk soil (BS) and soil adhered to peridium (SP) and the two truffle compartments of peridium (P) and gleba (G) (Table 2).

**FIG 4:**
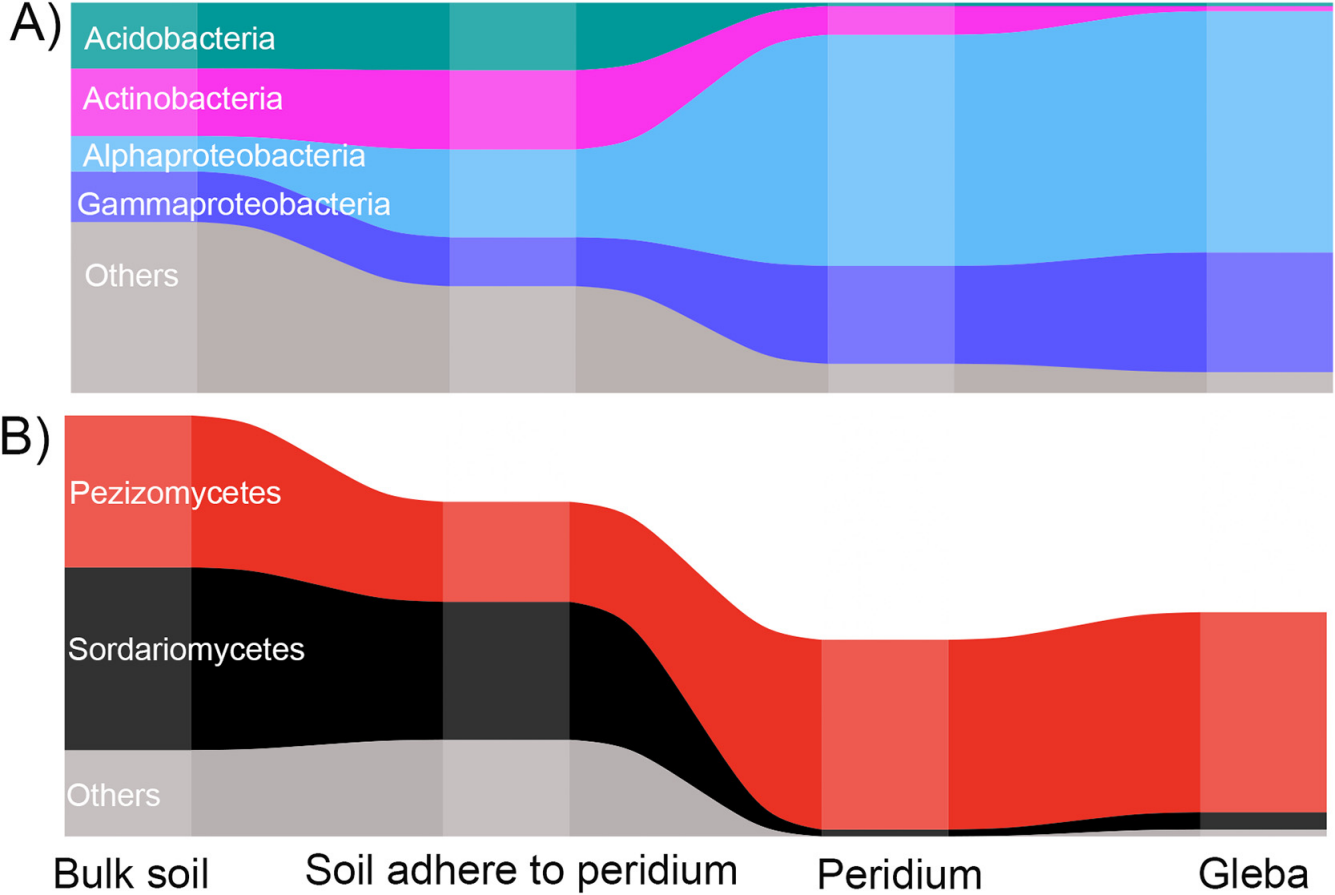
Focused alluvial diagram of bacteria (A) and fungi (B) in truffle-associated compartments. Each column represents a compartment. The flows among compartments represent the redistribution of OTU clusters that involved in network functional modules. To avoid color saturation, only dominant microbial taxa are colored.

In terms of the competitive/cooperative relationships, bacterial taxa exhibited a co-occurrence pattern, with positive correlations accounting for >60% of potential interactions observed in the ecological networks of four compartments (Fig. 2). In addition, a stronger tendency of positive/co-occurrence associations was found within truffles (∼72% positive links) than in soil (average, 65% positive links). In contrast, for fungal taxa, competitive relationships were identified in the interface between soil and truffle, including 65 and 52% negative links in the soil adhering in the peridium (SP) and peridium (P) networks (Fig. 3), whereas with 61 and 62% positive links in bulk soil and gleba networks, respectively (Fig. 3). In order to depict the microbial variation of the interphase established from the bulk soil to the inner truffle, we visualized those changes at phylum and/or class level by alluvial diagrams (Fig. 4). The bacterial taxa reallocation in dominating (six largest) modules occurred extensively at the interphase between soil and truffle, especially for the following three phyla: an obvious expansion for *Proteobacteria* and a strong exclusion for *Acidobacteria* and *Actinobacteria* (Fig. 4A), and such a trend was also observed for the individual network modules. For fungi, the number of modules clearly decreased from the bulk soil to the truffle gleba (Fig. 3). The interphase that links the outer and inner parts of the truffle substantially excluded the *Sordariomycetes* while *Pezizomycetes* (nonhost) were highly persistent (Fig. 4B). Moreover, we determined the topological role of individual OTUs in the bacterial and fungal networks consisting of all truffle-associated samples via the random matrix theory-based network analyses (Fig. 3).

**FIG 5:**
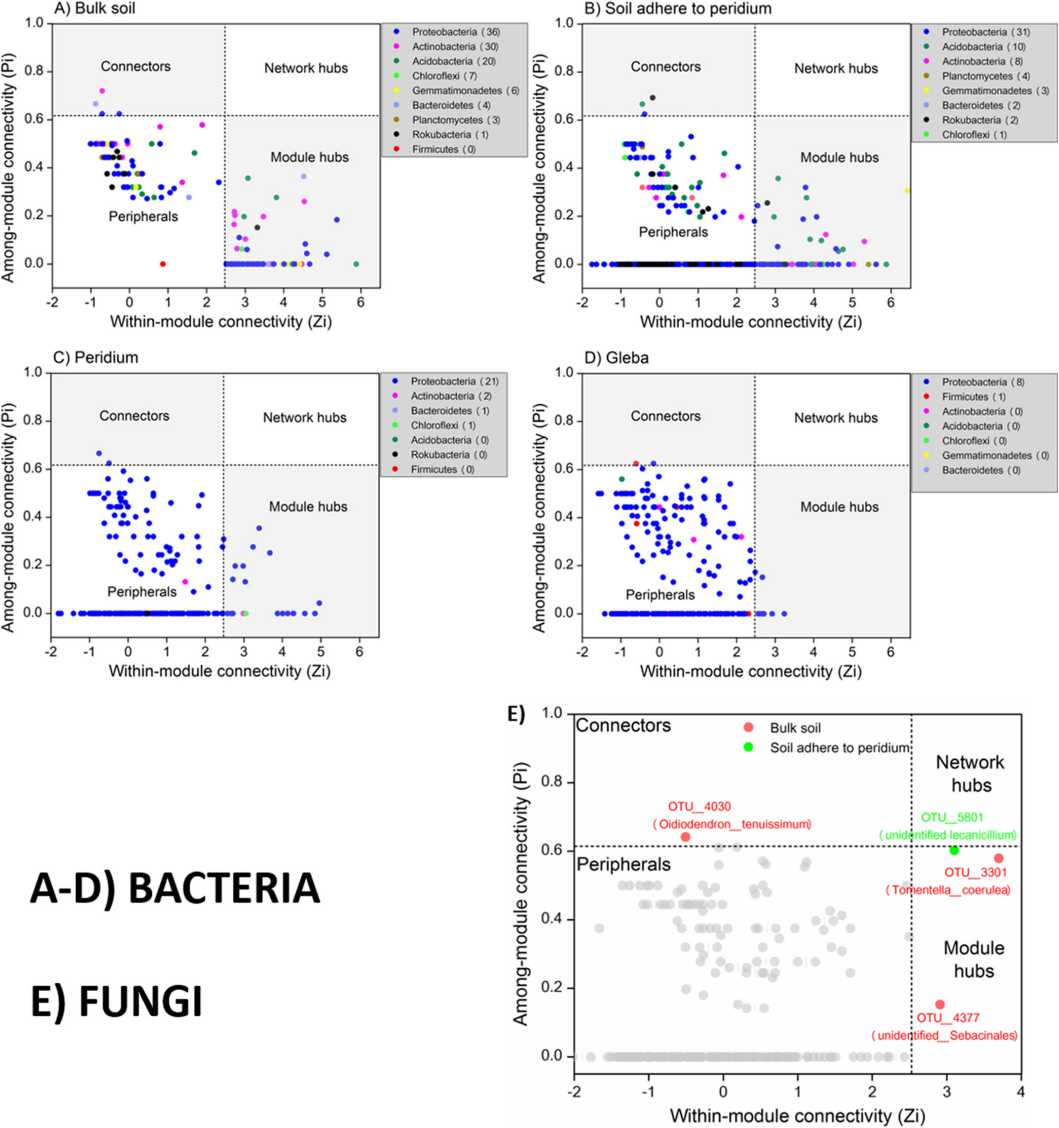
Z-P plots showing the classification of nodes to identify potential keystone bacterial (A to D) and fungal (E) species in four truffle-associated compartments. Each point represents an OTU. The topological roles of individual OTUs were determined based on scatterplots of within-module (Zi) and among-module (Pi) connectivities. The module hubs (Zi > 2.5) and connectors (Pi > 0.62) are highlighted as a gray-shaded area for bacteria (A to D) and as color points for fungi (E). For bacteria (A to D), color represents OTUs assigned to phyla, and the values in parentheses indicate the total numbers of keystone species (including module hubs and connectors). For fungi (E), no keystone taxa were detected in truffle peridium and gleba, whereas there were three keystone taxa found in bulk soil (BS) and one in soil adhered to the truffle peridium (SP) which are labeled with OTU numbers and species name.

No network hubs (supergeneralists) were detected in the four bacterial networks. Most (>90%) of the OTUs were peripherals with the majority of their links inside their modules. The numbers of bacterial keystone taxa (including both module hubs and connectors) at the phylum level decreased from soil (*n* = 8), to peridium (*n* = 4), to gleba (*n* = 2). Similarly, the numbers of keystone taxa decreased dramatically from soil to truffle: bulk soil (*n* = 107), to soil adhered to peridium (*n* = 61), to peridium (*n* = 25), to gleba (*n* = 9). The 107 bacterial keystone taxa in the bulk soil were dominated by *Proteobacteria* (*n* = 36), *Actinobacteria* (*n* = 30), and *Acidobacteria* (*n* = 20), while the rare taxa belonged to *Chloroflexi* (*n* = 7), *Gemmatimonadetes* (*n* = 6), *Bacteroidetes* (*n* = 4), *Planctomycetes* (*n* = 3), and *Rokubacteria* (*n* = 1). In contrast, the other three compartments were overwhelmingly occupied by *Proteobacteria* (31/66, 21/25, and 8/9) (Fig. 5B to D). For fungi, the keystone species were *Oidiodendron*, *Tomentella*, and *Sebacinales* in bulk soil (BS) and *Lecanicillium* in the peridium-adhering soil (Fig. 5E), but no keystone taxa were observed in the truffle peridium and gleba.

## DISCUSSION

Distinct microbiomes develop in forest ecological niches such as the phyllosphere (40, 41), roots (42), ectomycorrhize (34), and fruiting bodies (43, 44). Constitutionally, the niches that microbes inhabit and the substrates that microbes consume are the most important inherent factors shaping host-microbiome interactions. Thus, we proposed that such a “substrate-determinative” hypothesis could be proper in a narrow ecological niche such as that of the truffle-associated microbiome. Four typical compartments of the bulk soil, interphases (soil adhered to peridium, and peridium) and gleba tissue were chosen for testing our hypotheses regarding to the effect of host and compartment on a truffle microbiome’s diversity and ecology network changes.

In accordance with our first hypothesis (H1), the OTUs assigned to bacteria were substantially higher than those assigned to fungi in the four truffle-associated compartments (Fig. 1), and such dominances extended to truffle’s ascomata (Fig. 1). Truffle biology studies have revealed bacteria associated with aroma formation (12, 13), maturation (7), and nutrient fixation (7, 14). In our case, we found that there were 308 unique bacterial OTUs, whereas there were 78 unique fungal OTUs in the truffle gleba (see Fig. S2). Among truffle-associated compartments, soil harbored more complex microbiome compared to truffle interior (Fig. 2 and 3). In addition, we considered two microniches (truffle peridium and adhering soil to peridium) which are in close contact with the truffle inner gleba. We hypothesized that due to a clear microbiome exclusion existing in the interphase, microbiome diversity might be gradually decreasing from soil to inner truffle tissue. The results support this hypothesis (H2); there was a clear selection of microbiome by soil and peridium exemplified by a significant decrease in the alpha-diversity (Fig. 1). Spatially, the microbial diversity, evenness, and richness all decreased successively from the bulk soil, adhering soil to peridium, and peridium to gleba (Fig. 1). Ascomata have a physical and biochemical composition which is completely different to that of the surrounding soils. These specific conditions might be a deterministic factor of paramount ecophysiological relevance that strongly filter certain bacteria from the surrounding soils and allow them to proliferate in the truffle tissues due to the symbiotic or environmental requirements of ascomata-endophytic bacteria in ascomata (45). Physical and chemical changes include, for example, pH, temperature, oxygen levels, organic carbon availability, and ammonium released during chitin degradation in the course of fruit bodies maturation process, which may be selective factors for certain bacterial groups (46, 47). At the same time, the soil surrounding the ascomata constitutes a reservoir of biodiversity, from which specific bacterial communities are differentially recruited both in the truffle compartments and in the ectomycorrhizosphere. Bacterial communities associated with the inner ascomata compartments might play symbiotic and functional roles in their life cycle. Antony-Babu et al. (45) found functional potentials related to nitrogen fixation, cellulose and chitin degradation, and sulfur metabolism. Interestingly, previously N-fixation has been detected in the white truffle *Tuber magnatum* (35). Bacterial taxa involved in cellulose and chitin degradation, such as *Bacteroidetes* and Actinomycetes, contribute to the release of the ascospores; pseudomonads and spore-forming *Bacillaceae*, with proven chitinolytic and cellulolytic activities may play an important role in the ascus opening and spore scattering; and bacteria linked to sulfur metabolism contribute to the synthesis of aromatic volatile compounds containing sulfur, responsible for the characteristic truffle aroma. In addition, bacteria also may play important roles for fruiting bodies, such as inhibiting pathogens and antagonists, improving the distribution of spores, and providing vitamins and growth regulators (13, 43, 48, 49).

Notably, these results were not only obtained from a single host tree but from three typical truffle symbiont host trees (*P. armandii*, *P. yunnanensis*, and *Q. franchetii*) (Fig. 1). In contrast, there was not a clear influence of host rhizosphere on microbiome alpha- and beta-diversity (Fig. 1 and 2). The ectomycorrhizal mycelia of some fungi are sensitive to changes to host tree leaf litter composition (50). In contrast, our study shows that soils conditioned by different host trees have no significant influence in the ascomata microbial diversity (Table 1 and Fig. 2). In support of our third hypothesis (H3), the truffle microbiome composition was strongly driven by its associated compartments rather than by their symbiotic host trees (Table 1 and Fig. 2 and 3). In addition, the truffle-inhabiting bacterial community had more variation than those of the fungal community. There was significant higher bacterial diversity in truffle-associated compartments than those of for fungi (Fig. 1). Studies showed that the change in bacterial assembling structure ought to be closely related to their roles in the sensitive biological processes of nutrients exchange among soil, truffle tissue, and their interphases (48). This aforementioned interphase microbiome exclusion shows that there is a selection of soil microorganisms that can grow in the truffle peridium and from there colonize the inner gleba tissue (Fig. 4).

In the present study, we initially identified the core microbiome, the common OTU number was around six times higher for bacteria than for fungi (995 versus 157), which was in line with substantial differences between bacterial and fungal OTU richness supporting the fourth hypothesis (H4). Such differences might be explained by the microbiome differences in the initial truffle developing stage in ectomycorrhizospheric soil, because Li et al. found that the number of bacterial species was ten times higher than that of fungal species in ectomycorrhiza (1,514 versus 100), and six times in ectomycorrhizospheric soil (1,350 versus 181) (1). We further distinguished the variations on core microbiome among truffle-associated compartments. In gleba, the core bacterial OTUs were dominated by *Proteobacteria* (see Fig. S3). Similar results were found by Benucci et al. (44), who compared truffle microbiomes on gleba tissue from eight truffle species. The particularly abundant *Bradyrhizobium* and *Sphingobium* inside the gleba might reflect their potential roles in nitrogen fixation, as well as in glucose and fructose fermentation, respectively (35, 51, 52). In contrast to its function in carbon cycling (7) and ascospore release (53), *Bacteroidetes* predominated in the peridium. Notably, four bacterial phyla (*Rokubacteria*, *Nitrospirae*, *Chloroflexi*, and *Planctomycetes*) were recorded only in the surrounding soils, but they did not colonize the truffle ascomata (see Fig. S3), supporting the aforementioned interphase-excluding hypothesis. Interestingly, *Planctomycetes* was also abundantly found in soils but not in basidiomata belonging to the *Agaricales* genera: *Amanita*, *Lactarius*, *Paxillus*, and *Russula* in eastern Estonia (47). In addition, similar results have been reported in some *Basidiomycetes*, where the bacterial community structures differ between internal and external parts of the fruiting body but not between inner tissues (46). For fungi, our results demonstrated that (i) the Basidiomycota were dominant in soil, and the Rozellomycota tended to live in soil adhered to the truffle peridium, that is, a clear shift from Basidiomycota- to Rozellomycota-associated soils compared to truffle ascomata. The Rozellomycota (or Cryptomycota) is a newly described division of fungi, that is distributed in a diversity of habitats and geographical locations, including soils, marine and freshwater sediments, freshwater planktonic samples, and oxygen-depleted environments (54). Although their ecology is mostly unknown, a single-cell lifestyle frequently supports their occurrence in unusual environments. Globally, the Rozellomycota has been reported to be one of the most abundant fungal taxa in the soils (55). (ii) The number of fungal taxa with high relative abundances tended to increase from gleba (*n* = 3) to peridium (*n* = 6). *Phaeoacremonium* was one of the six most abundant genera in the truffle peridium (see Table S1), which could be a great potential contributor to the early peridium formation since it is enriched in the ectomycorrhizae of the same truffle species (1). The potential interactions of individual taxa within truffle microbiome were explored, and we further investigated the response of the whole microbiome to external changes as a “network” (Fig. 2 and 3). Considering the higher bacterial richness and diversity evaluated via bacterial OTU number and the fact that their associations would be more complex, we hypothesized that the complexity of bacterial and fungal networks would gradually decrease from bulk soil to soil-truffle interphase and further to gleba tissue (H4). The results from the molecular ecological network analysis verified this hypothesis (Table 2). Our data support that the complexity of microbiome steadily weakened from bulk soil to soil-truffle interphase and further to gleba tissue. Such “ecological filtering” could be explained by (i) the influence of substrate and its screening on residing microbes, as reflected by the distinctly varied microbial OTUs in compartments; (ii) positive links between phylogenetic richness and the strength of their significant relationships—with more significant correlations existing in a compartment harboring high microbial richness; and (iii) modularity discrepancy among compartment—with more functional modularity of microbiome performing nutrient cycling and organic matter degradation, etc. (33, 56), in soils than in truffles (Fig. 2 and 3). These network complexities could deepen our better understanding of internal characteristics or interactive dynamics of microbial communities. Based on the OTU-level variables (e.g., average clustering coefficient and connectivity), evaluation of the network aggregate/loose structure is possible to demonstrate that the truffle microbiome networks, rather than soil microbial networks, harbored closer and interconnected microbial OTUs (Table 2). Furthermore, we estimated the taxa competitive/cooperative relationship between individual networks and found that bacterial OTUs exhibited a co-occurrence (in comparison to fungi; 56% negative links). The positive co-occurring trend was higher in truffles (∼72% positive links) than in soil (∼65% positive links; Fig. 2 and 3), indicating that a stronger aggregated microbial network was observed in the truffle-inhabiting microbiome than in the soil microbiome. Recent studies have shown that microbial communities harbor keystone taxa (irrespective of their abundance), which drives microbiome composition and functioning (29, 57). In line with the changes in network complexity, the number of keystone taxa from random matrix theory-based network analysis dramatically decreased from soil to truffles. The abiotic and biotic factors, including, for example, nutrient and microbial composition, among the evaluated truffle microniches might explain the recorded changes prevailing between soils and truffles. Here, the bulk soil harbored the most complex microbiome network and the highest number of keystones (*n* = 107) and, accordingly, the highest keystone diversity (taxa assigned to eight bacterial phyla). This indicates that multiple keystone taxa (i.e., 107) in bulk soil and 61 in soil adhered to the truffle peridium (Fig. 5) might form a keystone guild and could influence a broadly microbial process, such as organic matter decomposition and denitrification (28), whereas in truffles the keystone taxa were almost assigned to only one dominating proteobacterial phylum that could reflect certain N-fixing bacteria (such as *Bradyrhizobium* and *Cupriavidus*; see Table S3) or functioned alone within truffle fruiting bodies. This indicates that truffle keystone taxa tended to have stronger effects on a relatively narrow process such as nitrogen and sulfur cycling (7, 35). For fungi, no keystone taxa were detected in truffle peridium and gleba; there were only three keystone taxa found in the bulk soil, and one in soil adhered to the truffle peridium (Fig. 5E), showing their weak involvements as connectors and module hubs with a community. Although network analysis can be used to computationally identify keystone taxa in microbial networks, it is important to link such taxa to ecosystem processes. For the next step, it is a challenge to complement theoretical evidence with empirical evidence (RNA-stable isotope probing coupled with metaproteomics or metatranscriptomics) to identify keystone taxa in microbial communities.

10.1128/mSphere.00039-21.6 TABLE S3 Potential keystone species (module hubs) in the *Tuber indicum* gleba compartment. Zi, within-module connectivity; Pi, among-module connectivity. Download Table S3, DOC file, 0.05 MB. Copyright © 2021 Liu et al. 2021 Liu et al. https://creativecommons.org/licenses/by/4.0/ This content is distributed under the terms of the Creative Commons Attribution 4.0 International license.

### Outlook and conclusion.

We conclude that the truffle-associated compartments, rather than the host trees, are a more important driver to construct the bacterial and fungal communities associated with *Tuber indicum* ascomata. This study highlights for the first time the multiple and complex potential interactions between bacterial and fungal associations in truffles. Since this study was conducted in a single location, it is possible to draw robust conclusions due to the fact that biotic and abiotic variations were the same for our treatments. However, further studies involving comparisons between different locations would provide deeper and more valuable insights, showing whether the results are similar regardless of the environment considered or site dependent. Another promising perspective would come from assessing the transcriptomic activities of microbial community associated with truffles and to determine the functional profiles and metabolic pathways related to truffle cultivation at a global scale in order to increase the productivity of these profitable highly prized fungi.

## MATERIALS AND METHODS

### Sampling and storage.

This research was conducted in the Puding Karst Ecological Experimental Station (Puding, Guizhou, 26°54′59.7″N, 105°42′35.5″E, 1,325 m above the sea level), belonging to the Institute of Geochemistry, Chinese Academy of Sciences (CAS). *Tuber indicum* was cultivated with three different host trees (*Quercus franchetii*, *Pinus armandii*, and *P. yunnannensis*) in orchards covering 3,000 m^2^ each one. Inside these orchards, three 50 × 50 subplots, containing at least six individual trees, were selected for biological replicate sampling in December 2018. From 10 to 20 mature truffles, with no insect larvae galleries or animal injures, were collected from each of the three subplots, and three high-quality, intact, and healthy truffles were chosen for truffle-associated compartments tissue separation. Bulk soil (BS) was also collected 5.0 m away from the tree trunk to avoid the influence of the truffle’s sphere. Soils adhered to the peridium (SP) were those tightly adhering on the truffle surface (<0.5 cm). SP was collected on a sterile petri dish with a sterilized soft-metal brush and then transferred into a 5.0-ml tube. After soil collection, all truffles were cleaned with sterilized Milli-Q water and dried with sterilized absorbent paper. The ascomata were then cut using sterilized scalpels, and the truffle peridium (P) and gleba tissues (G) were collected by using sterilized forceps. The respective compartments of G and P from five truffles were mixed together and stored in alcohol sterilized self-sealing bags (60 mm × 85 mm) as a composite sample at −20°C for subsequent species identification (ITS and SSU) and DNA extraction. The experimental design was a two-factor design (3 host trees × 4 compartments = 12 treatments), with three biological replicates per treatment. Thus, 36 samples were evaluated in total.

### DNA extraction and PCR amplification.

Two extraction methods were used to isolate microbial DNA from truffle and soil samples. Soils (BS and SP) were extracted using the MoBioPower Soil DNA kit (catalog no. 12888), and truffle samples were processed using a DNeasy plant minikit (Qiagen SA). PCR amplifications were carried out according to a previously described method (58). Briefly, a 25-μl reaction mixture was set up containing 5 μl of 5× reaction buffer, 5 μl of 5× GC buffer, 2 μl of deoxynucleoside triphosphate (2.5 mM), 1 μl of reverse primer (10 μM), 1 μl of forward primer (10 μM), 8.75 μl of ddH_2_O, 2 μl of DNA template, and 0.25 μl of Q5 DNA polymerase. The V4 hypervariable region of the bacterial 16S rRNA gene was amplified using two primers (338F and 806R) as described by Mori et al. (59). Internal transcribed spacer 1 (ITS1) was used for fungal identification, with the primers ITS5F and ITS1R (60). Negative controls containing all PCR reagents except template DNA and ZymoBIOMICS positive controls were used. The PCR thermal cycling conditions were as follows: 98°C for 2 min (initial denaturation); 25 to 30 cycles of 98°C for 15 s, 55°C for 30 s, and 72°C for 30 s; with a final extension for 5 min at 72°C. Amplicons were extracted from 2% agarose gels, purified with an Axygen Axy Prep DNA gel extraction kit (AP-GX-500) according to the manufacturer’s guidelines, and quantified by the Quant-iT PicoGreen dsDNA assay kit (Invitrogen, P7589) on a microplate reader (BioTek, FLx800).

### Illumina MiSeq sequencing and bioinformatics.

Purified amplicons were pair-end sequenced 2 × 300 on Illumina MiSeq platform (MiSeq-PE250; Personalbio, Shanghai, China) using a MiSeq reagent kit v2 (600-cycles-PE, MS-102-3003). Sequences were processed and quality filtered using the QIIME (Quantitative Insights into Microbial Ecology) pipeline. The 300-bp reads ends were truncated from the first site with low quality (average quality value <20 over a 10-bp sliding window, and the undesirable truncated reads (length < 150 bp) were filtered. Then, the overlapping sequences (≥10 bp and passed through quality screening) were assembled using the FLASH software (v1.2.7) (61). After chimera detection, the obtained high-quality sequences were processed with USEARCH software (http://www.drive5.com/usearch/). The SILVA (for 16S, https://www.arb-silva.de/) and Unite (for ITS, http://unite.ut.ee/index.php) databases were used to annotate taxonomic information (62, 63). OTUs with an abundance of <0.001% of the total sequences across all samples were removed (61) from the final analysis. The taxonomic cutoff was set at a 97% similarity for genus level, and OTUs assigned to same phylum, class, order, family, and genus level were grouped together based on their taxonomic affiliations.

### Data processing and statistical analysis.

For each bacterial and fungal community inhabiting in various truffle-associated compartments from different host trees, the Chao 1 richness index was calculated from the total OTUs, as well as for the two diversity indices, i.e., Shannon index (which considers both the numbers of individual taxa and of OTUs; Fig. 1) and Simpson evenness index (a measure of the relative abundance of the different individual taxa making up the richness of an area/sample).

The microbial alpha-diversity was estimated by richness (Chao 1) and diversity (Shannon and Simpson) indexes. One-way analysis of variance, followed by Tukey HSD (at *P < *0.05), was used to compare significant differences in diversity indices.

The beta-diversity values of the overall microbial communities between paired samples were determined using the UniFrac metric (66) in the MOTHUR program (http://www.mothur.org), and NMDS analysis was performed using the vegan package of R software based on weighted and unweighted UniFrac distance matrices, and the obtained points were plotted using Origin 2018.

We constructed the phylogeny of the bacterial OTUs (with a threshold of relative abundance at the top 100 in order to provide a relatively equal comparison and to reduce complexity of a tree) commonly shared by four compartments to assess (i) the phylogenetic selection of these OTUs and (ii) the domains of a compartment in individual species. For the bacterial data set, phylogenetic trees were constructed in QIIME using the maximum-likelihood method and visualized using the online Interactive Tree of Life.

### Network and keystone taxa analyses.

Individual networks were constructed for various compartments/host trees based on 16S rRNA or ITS1 gene sequence data. The molecular ecological network analysis (MENA) pipeline (http://ieg4.rccc.ou.edu/mena/help.cgi) was used to analyze the networks. In brief, there were four main steps for network constructions: (i) original data collection (OTU table), (ii) data standardization (with “lg” transformation), (iii) pairwise correlation/similarity estimation, and (iv) adjacent matrix formatting based on a random matrix theory method (24). Indices to evaluate the features of the nodes were as follows: (i) degree, a node with higher degree means that it is highly connected with other nodes (that is, high degree = strong relationship with others); (ii) betweenness centrality (BC) = among-module connectivity (Pi; the parameter was used to indicate nodes connecting modules [connectors], Pi > 0.62); (iii) closeness centrality (CC) = within-module connectivity (Zi), referring to highly connected nodes within modules (module hubs), Zi > 2.5); (iv) important nodes to both the network and its own module coherence = network hub (Zi > 2.5, Pi > 0.62); and (v) peripherals—for nodes within the module but few outside connection, Zi < 2.5 and Pi < 0.62). The nodes with either a high value of Zi or Pi were defined as keystone taxa, including network hubs, module hubs, and connectors (64). The numbers of nodes, links, and modules were used to reflect the complexity of a microbial network, and the microbial interconnection within a network was evaluated by the node-based variables such as averaged clustering coefficient and connectivity. To reveal stories in the large networks, we used alluvial diagrams to highlight and map the significantly structural changes in the network data (65). The microbial module data for each truffle compartment were obtained from the online platform MENA and were rearranged in an Excel file. Since the bacterial network contained a high number of modules (>100), we set two criteria to capture major information in the complex functional modules: (i) selecting the top six modules that had the highest number of highly interconnected OTUs and then (ii) filtering and keeping the same OTU numbers in individual modules. In this way, we could map the trend of changes in bacterial phyla participating in these major modules. For fungi, no selective criteria were needed, considering their limited number of modules. Detailed changes in bacterial and fungal diagrams were constructed and visualized with the ggplots2 package in R, based on the demonstrating link (https://cran.r-project.org/web/packages/ggalluvial/vignettes/ggalluvial.html). For network construction, the OTU tables were not limited to core taxa. We used the “OTU_even_depth” table, which was rarified according to 95% of the minimum sequence size of the sample.

### Data availability.

The sequences of 16S rRNA and ITS genes have been deposited in the NCBI Sequence Read Archive under accession number SRP126991.
