# Supplementary material for: Truffle Microbiome Is Driven by Fruit Body Compartmentalization Rather than Soils Conditioned by Different Host Trees
Source: mSphere. 2021 Aug 11;6(4):e00039-21. doi: 10.1128/mSphere.00039-21 (PMC8386477; doi:10.1128/mSphere.00039-21)
Supplement: FIG S1 [file msphere.00039-21-sf001.doc]

**
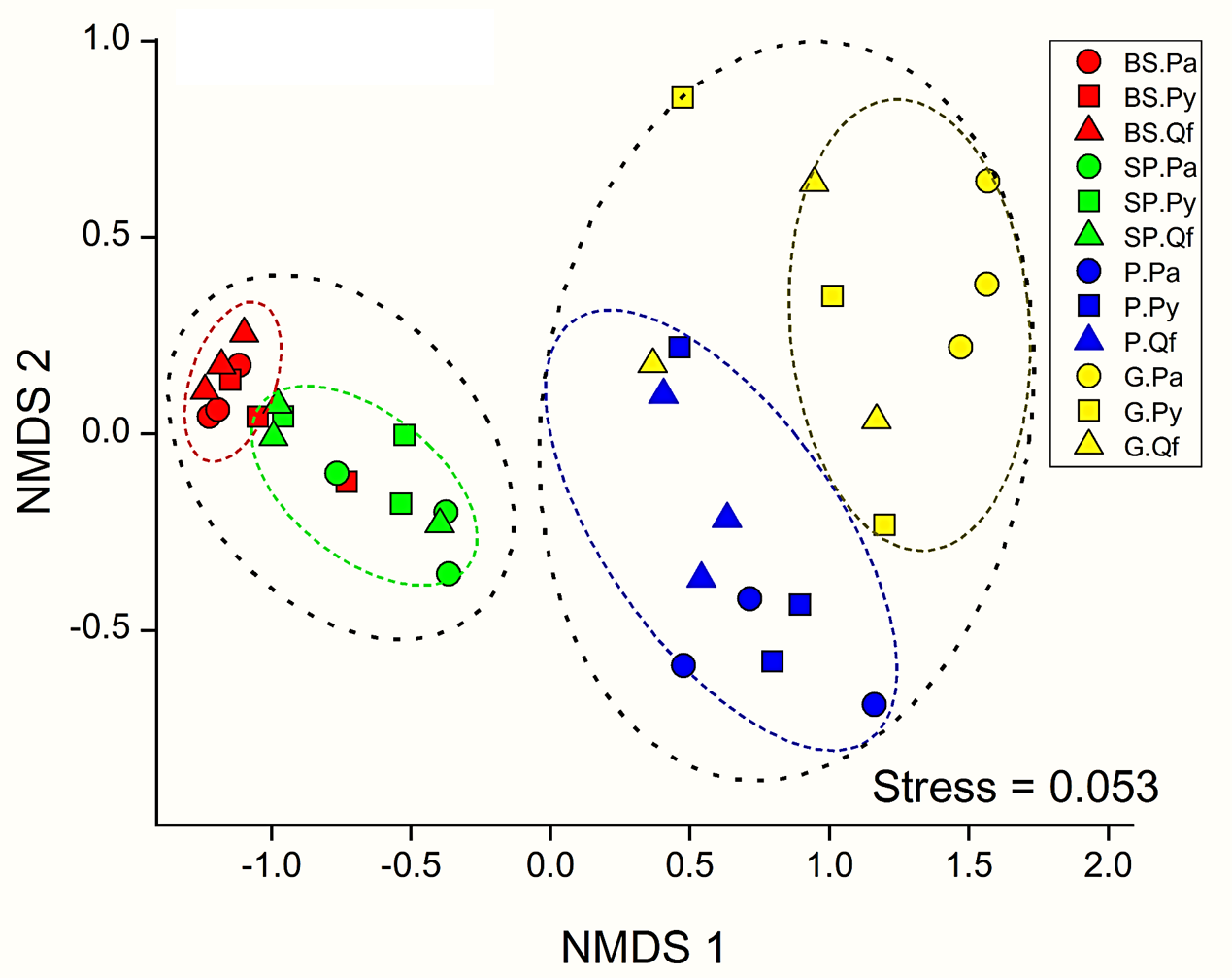
**

**Supplementary Figure S1** Bacterial community compositions as indicated by unweighted no-metric multi-dimensional scaling plots (NMDS) of pairwise UniFrac community distance across the *Tuber indicum* recorded in four different ascocarp compartments and three different host trees. Each abbreviation is composed of “compartment/soil conditioned by host tree”. For truffle compartments: BS = bulk soil; SP = soil adhered to the truffle peridium; P = peridium; G gleba. For soil conditioned by host trees: Pa = *Pinus* *armandii*; Py = *Pinus* *yunnanensis*; Qf = *Quercus* *franchetii*.
