# Supplementary material for: Truffle Microbiome Is Driven by Fruit Body Compartmentalization Rather than Soils Conditioned by Different Host Trees
Source: mSphere. 2021 Aug 11;6(4):e00039-21. doi: 10.1128/mSphere.00039-21 (PMC8386477; doi:10.1128/mSphere.00039-21)
Supplement: FIG S2 [file msphere.00039-21-sf002.doc]

**
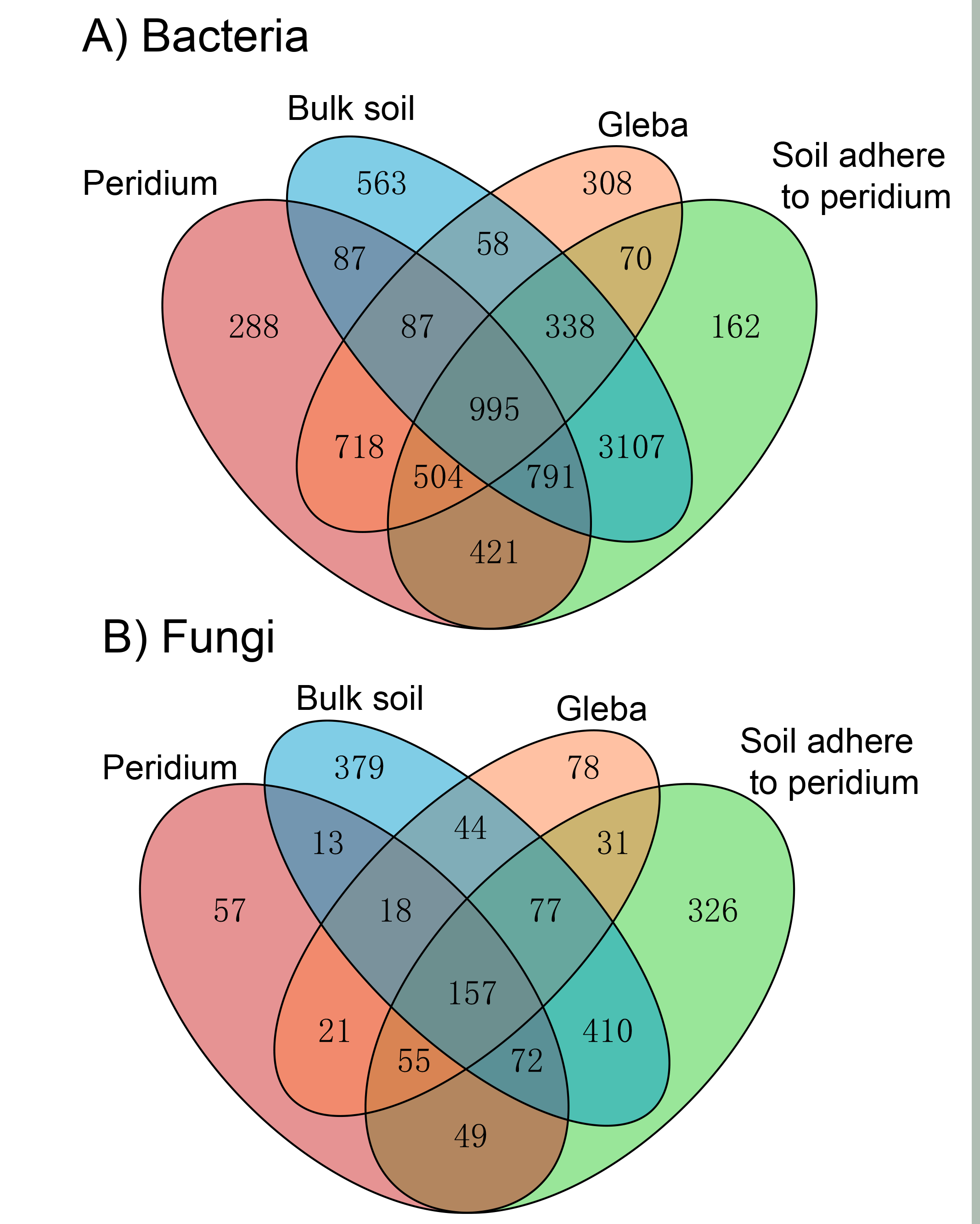
**

**Supplementary Figure S2.** OTU numbers of bacteria (A) or fungi (B) shared or exclusive of four *Tuber indicum* compartments (BS = bulk soil; G = gleba; P = peridium; SP = soil adhered to peridium). The total number of shared OTUs bacteria and fungi is shown in the center. The OTUs exclusively present in each compartment are shown in different colors as dark pink = peridium; blue = bulk soil; pink = gleba; and green = soil adhere to peridium, and all the possible intersections.
