# Supplementary material for: Truffle Microbiome Is Driven by Fruit Body Compartmentalization Rather than Soils Conditioned by Different Host Trees
Source: mSphere. 2021 Aug 11;6(4):e00039-21. doi: 10.1128/mSphere.00039-21 (PMC8386477; doi:10.1128/mSphere.00039-21)
Supplement: FIG S3 [file msphere.00039-21-sf003.doc]

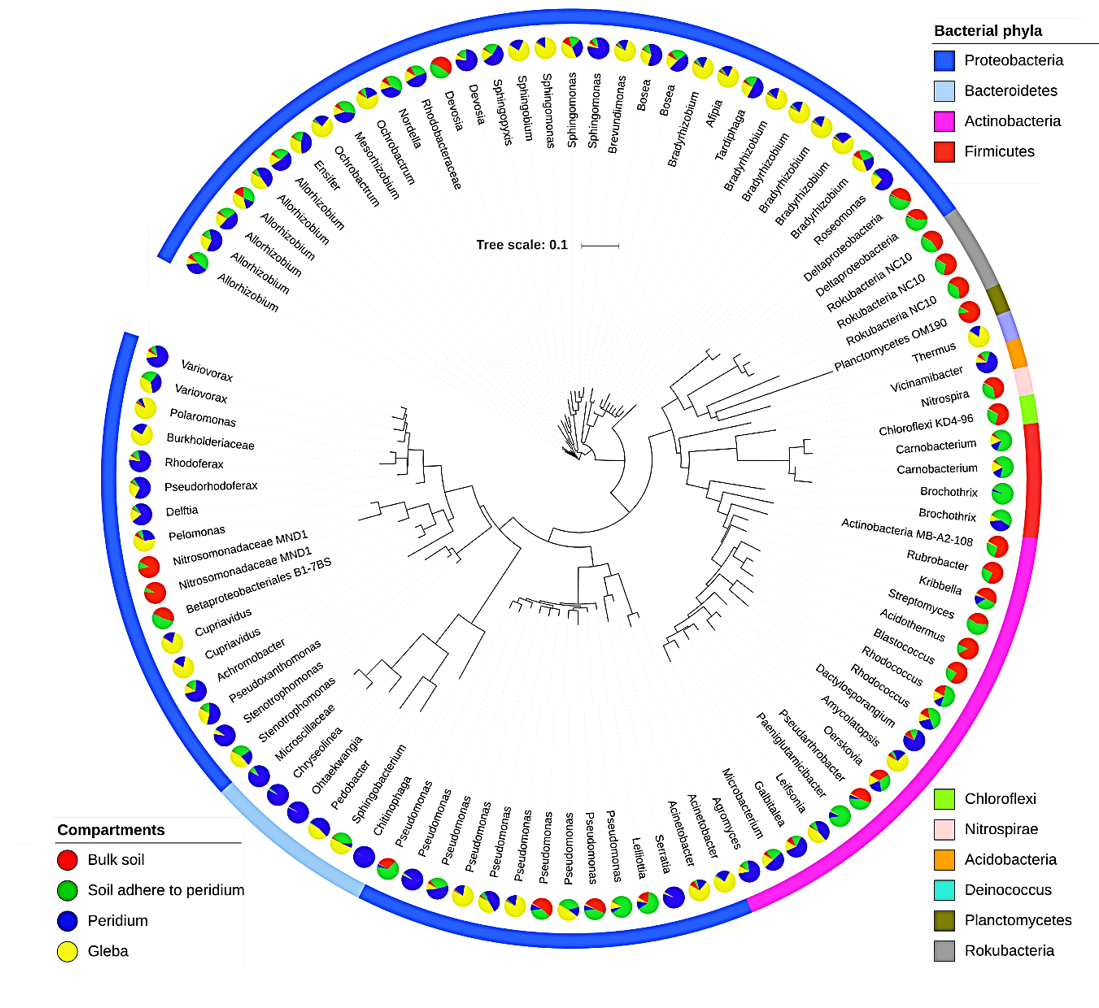


**Supplementary Figure S3.** Phylogenetic tree of OTUs common to the four truffle-associated compartments based on analysis of 16S rRNA genes. Colors of stripes indicate different major phyla. The pie charts represent the relative abundance of each OTU, and the pie slice colors the distribution across compartments (bulk soil in red, soil adhered to the truffle peridium in green, peridium in blue and gleba in blue).
