# Supplementary material for: Truffle Microbiome Is Driven by Fruit Body Compartmentalization Rather than Soils Conditioned by Different Host Trees
Source: mSphere. 2021 Aug 11;6(4):e00039-21. doi: 10.1128/mSphere.00039-21 (PMC8386477; doi:10.1128/mSphere.00039-21)
Supplement: TABLE S2 [file msphere.00039-21-st002.doc]

**Supplementary Table 2.** Relative abundance of the most abundant bacteria and fungi in *Tuber indicum* compartments.

| Taxon | Mean | Bulk soil | Soil adhered to peridium | Peridium | Gleba |
| --- | --- | --- | --- | --- | --- |
| k__Bacteria;p__Acidobacteria | 6.96% | 14.58% | 11.45% | 1.43% | 0.40% |
| k__Bacteria;p__Actinobacteria | 11.01% | 20.88% | 12.57% | 7.81% | 2.79% |
| k__Bacteria;p__Bacteroidetes | 7.24% | 2.19% | 4.57% | 13.77% | 8.45% |
| k__Bacteria;p__Chloroflexi | 3.31% | 7.16% | 4.77% | 1.03% | 0.29% |
| k__Bacteria;p__Deinococcus-Thermus | 0.12% | 0.01% | 0.00% | 0.08% | 0.39% |
| k__Bacteria;p__Firmicutes | 4.25% | 0.60% | 9.59% | 3.51% | 3.31% |
| k__Bacteria;p__Nitrospirae | 0.33% | 0.98% | 0.33% | 0.01% | 0.01% |
| k__Bacteria;p__Planctomycetes | 1.97% | 5.41% | 2.28% | 0.07% | 0.12% |
| k__Bacteria;p__Proteobacteria | 59.55% | 36.16% | 47.99% | 70.91% | 83.16% |
| k__Bacteria;p__Rokubacteria | 1.35% | 3.25% | 2.08% | 0.03% | 0.05% |
| k__Fungi;p__Ascomycota | 79.33% | 63.74% | 60.54% | 97.19% | 95.83% |
| k__Fungi;p__Basidiomycota | 9.71% | 24.04% | 11.96% | 1.30% | 1.54% |
| k__Fungi;p__Chytridiomycota | 0.61% | 0.37% | 2.00% | 0.05% | 0.01% |
| k__Fungi;p__Mortierellomycota | 1.07% | 2.47% | 1.72% | 0.01% | 0.07% |
| k__Fungi;p__Mucoromycota | 3.65% | 2.54% | 11.85% | 0.07% | 0.13% |
| k__Fungi;p__Rozellomycota | 0.41% | 0.45% | 1.01% | 0.01% | 0.16% |
