# Supplementary material for: Truffle Microbiome Is Driven by Fruit Body Compartmentalization Rather than Soils Conditioned by Different Host Trees
Source: mSphere. 2021 Aug 11;6(4):e00039-21. doi: 10.1128/mSphere.00039-21 (PMC8386477; doi:10.1128/mSphere.00039-21)
Supplement: TABLE S3 [file msphere.00039-21-st003.doc]

**Supplementary Table 3.** Potential keystone species (module hubs) in the *Tuber indicum* gleba compartment. Zi, within-module connectivity; Pi, among-module connectivity.

| Node Name | Phyla | Genus | Zi | Pi |
| --- | --- | --- | --- | --- |
| OTU 39706 | Proteobacteria | *Acinetobacter* | -0.15 | 0.625 |
| OTU 20425 | Proteobacteria | *Aminobacter* | 2.921 | 0 |
| OTU 81703 | Proteobacteria | *Bradyrhizobium* | 2.669 | 0.152 |
| OTU32236 | Proteobacteria | *Cupriavidus* | 3.236 | 0 |
| OTU 54799 | Proteobacteria | *Cupriavidus* | 2.687 | 0 |
| OTU 94614 | Proteobacteria | *Cupriavidus* | 2.562 | 0 |
| OTU 35428 | Firmicutes | *Cupriavidus* | -0.605 | 0.625 |
| OTU 32392 | Proteobacteria | *Mesorhizobium* | 2.707 | 0 |
| OTU 61272 | Proteobacteria | uncultured_bacterium | 2.645 | 0 |
